# Afraid of Microbes

**Published:** 1897-11

**Authors:** 


AFRAID OF MICROBES.—As an indication of the feverish apprehension existing in the country, lest yellow fever should pay them another visit, the constable of Precinct No. 2, ......... County, wrote the State Health Officer, asking if he could not make certain parties in New Orleans,—giving the name of a commission firm,—stop sending circulars to his friends, Messrs. B...... & H......, the village butchers. The constable was politely informed that the authority of the Health Officer hardly extended that far; that the privilege of sending circulars was one of those privileges guaranteed to every citizen by the great bill of personal rights (provided he pays the postage), but that there is nothing in the bill of rights, nor of wrongs, either, as to that, that compels a man to take the circulars out of the office; —his rights in the matter consisting of the right of refusal. He was assured, if afraid of microbes, that all New Orleans mail matter is fumigated and rendered harmless.

